# Supplementary material for: Bidirectional Underwater Drag Reduction on Bionic Flounder Two-Tier Structural Surfaces
Source: Biomimetics (Basel). 2023 Mar 11;8(1):116. doi: 10.3390/biomimetics8010116 (PMC10046520; doi:10.3390/biomimetics8010116)
Supplement: Supplementary file 1 [file biomimetics-08-00116-s001.zip › biomimetics-2251631-supplementary.pdf]

biomimetics

MDPI

Article

Bidirectional Underwater Drag Reduction on Bionic Flounder Two-Tier Structural Surfaces

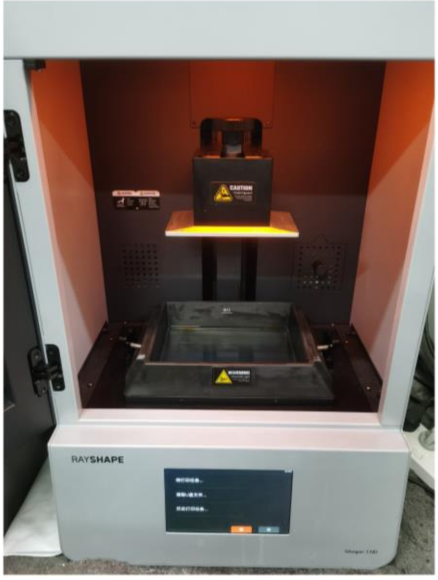

Figure S1. Image of 3D printer.

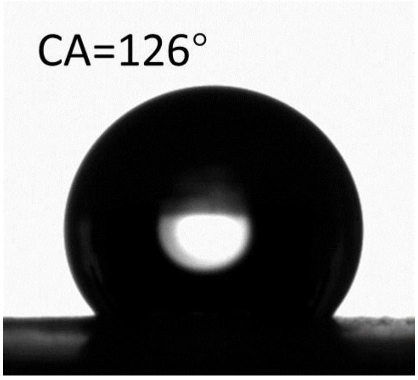

Figure S2. Contact angle of the resin after curing.

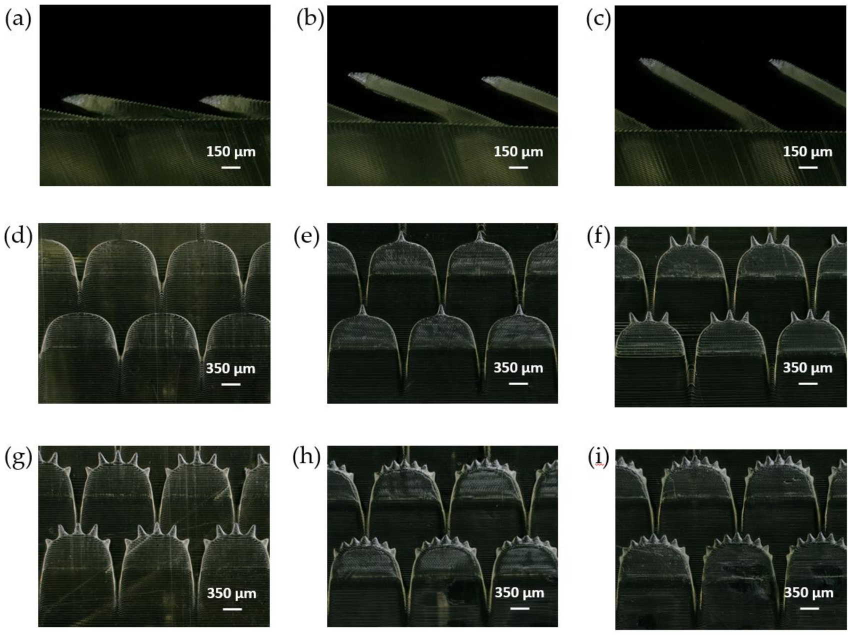

Figure S3. The test samples with different α and N: (a ~ c) different α, (d ~ i) different N.

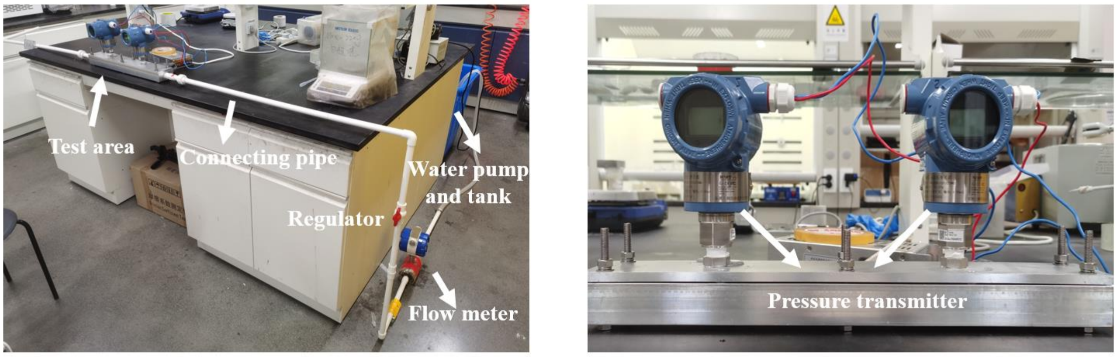

Figure S4. Self-built differential pressure resistance test platform.

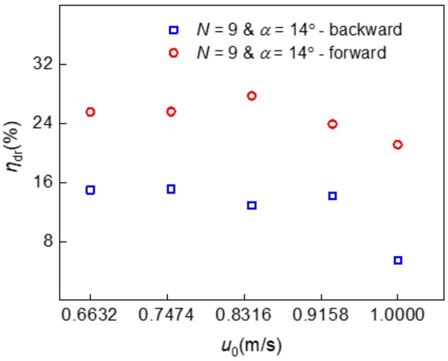

Figure S5. Drag reduction at different speeds with N = 9 and α = 14°.

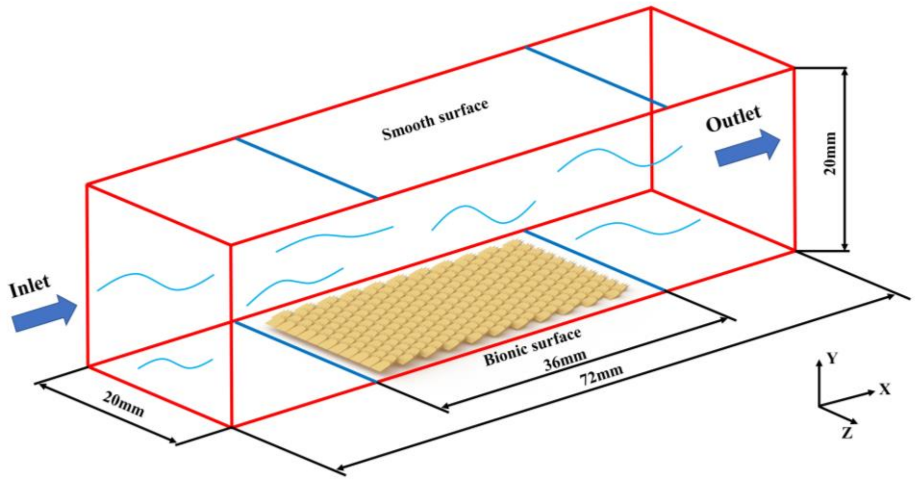

Figure S6. Biomimetic surface model for drag reduction experiments simulated by simulation software.

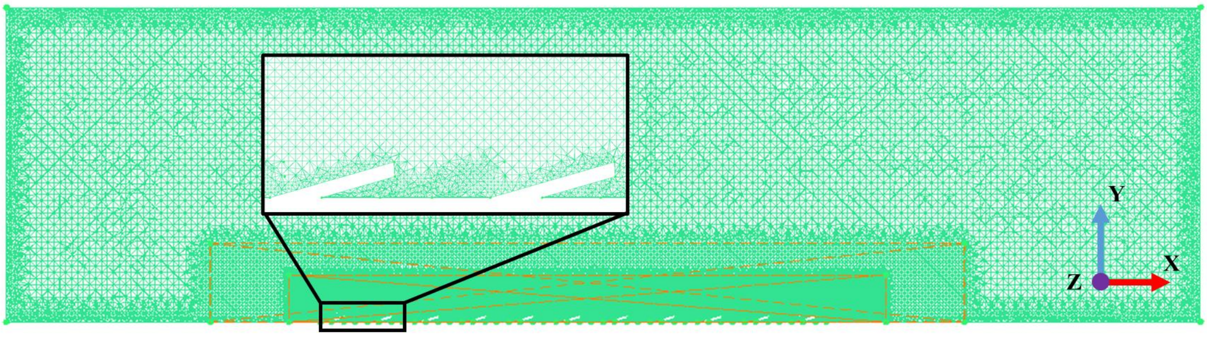

Figure S7. Meshing of the computational domain.

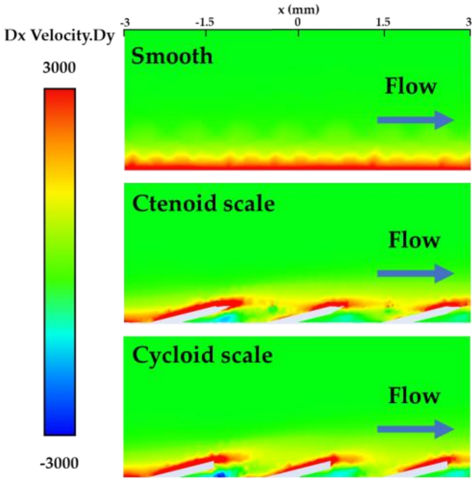

Figure S8. Velocity gradient diagram for the near-wall region on different surfaces.
